# Pretreatment levels of the serum biomarkers CEA, CYFRA 21–1, SCC and the soluble EGFR and its ligands EGF, TGF-alpha, HB-EGF in the prediction of outcome in erlotinib treated non-small-cell lung cancer patients

**DOI:** 10.1186/s40064-015-0891-0

**Published:** 2015-04-09

**Authors:** Elena Yaiza Romero-Ventosa, Sonia Blanco-Prieto, Ana Lourdes González-Piñeiro, Francisco Javier Rodríguez-Berrocal, Guadalupe Piñeiro-Corrales, María Páez de la Cadena

**Affiliations:** Hospital Pharmacy Service, Complejo Hospitalario Universitario de Vigo (CHUVI), 36204 Vigo, Spain; Department of Biochemistry, Genetics and Immunology, Faculty of Biology, University of Vigo, 36310 Vigo, Spain; Pathological Anatomy Service, Complejo Hospitalario Universitario de Vigo (CHUVI), 36204 Vigo, Spain

**Keywords:** Erlotinib, EGFR, CEA, Non-small cell lung cancer, Survival prediction, Prognosis

## Abstract

The aim of this study has been to investigate the potential of serum biomarkers used in clinical practice (CEA, CYFRA 21–1, SCC) together with the serum epidermal growth factor receptor (EGFR) and its associated ligands (EGF, TGF-α, HB-EGF) as outcome predictors of non-small cell lung cancer (NSCLC) patients treated with the TKI erlotinib. The pretreatment levels of these markers were evaluated through immunoassays carried out in 58 patients. The progression-free survival (PFS) and overall survival (OS) were assessed by the Kaplan-Meier method and differences between groups were compared by means of the Log-Rank test. Association of risk factors with survival was evaluated using the univariate and multivariate Cox modelling procedures. Higher CEA (>5 ng/mL) and sEGFR (>56.87 ng/mL) concentrations associated significantly with a higher overall survival. The pre-treatment sEGFR serum levels constituted an independent prognostic factor. The *EGFR* gene mutational status and the sEGFR level combination was the single to associate significantly with longer progression-free survival periods, in circumstances in which the *EGFR* gene was mutated and increased protein serum levels were detected. The overall survival as assessed through a Cox analysis revealed similar death hazards with respect to low sEGFR levels combined both with non-mutated *EGFR* genotypes and low CEA serum levels. Our results suggest that the pre-treatment CEA and sEGFR serum levels may provide a comparable source of information to that supplied by the *EGFR* gene mutational status with respect to the prognosis of erlotinib treated NSCLC patients. A combined sEGFR and CEA level appraisal could be of considerable value to select patients to undergo EGFR-TKI treatments.

**Electronic supplementary material:**

The online version of this article (doi:10.1186/s40064-015-0891-0) contains supplementary material, which is available to authorized users.

## Introduction

Non-small cell lung cancer (NSCLC) cases account for approximately 85% of all the lung cancer instances, with pulmonary carcinomas representing worldwide the leading death cause derived from cancer (Jemal et al. 2010). The uncovering of the epidermal growth factor receptor (EGFR) responsible for cell proliferation and survival (Baselga 2002) as being constitutively over-expressed in the majority of these tumour types, prompted the development of a number of anti-EGFR agents for NSCLC treatment. The best known anti-EGFR agents comprise the EGFR tyrosine kinase inhibitors (TKIs), such as gefitinib or ZD1839 (Iressa®, 2005), else erlotinib or OSI-774 (Tarceva®, 2009), all of these agents functioning through the inhibition of the EGFR phosphorylation and tyrosine kinase activities as mediated by competitive binding processes (Ciardiello and Tortora 2008).

Initial clinical trials concluded that some clinical and pathological features (Asian ethnicity, never-smokers, female gender and adenocarcinoma histology) all benefited by displaying longer survival responses to EGFR-TKI therapy (Shepherd et al. 2005; Kim et al. 2008; Cappuzzo et al. 2007; Tokumo et al. 2005). Nowadays, it has been clearly established that the *EGFR* gene mutational status comprises a powerful predictor of the tumour responses to EGFR-TKI treatments (Lynch et al. 2004; Janne et al. 2005; Cappuzzo et al. 2005, Takano et al. 2005; Hirsch et al. 2006; van Zandwijk et al. 2007; Mitsudomi et al. 2010), in consequence being widely used to select patients likely to respond to the medication. Findings derived from the EURTAC (Rosell et al. 2012) and OPTIMAL studies (Zhou et al. 2011) have strengthened the rationale to apply prognostic mutation status checking in the case of NSCLC patients.

Nevertheless, inclusive among *EGFR* mutated patients, not all individuals respond to EGFR-TKI treatment in the same manner, as a result the objective positive response to EGFR-TKI treatment has ranged 62% to 75% (Mitsudomi et al. 2010; Tamura et al. 2008; Maemondo et al. 2010). On the other hand, no *EGFR* mutations were identified in 10-20% of patients with partial responses to EGFR-TKI application (Pao et al. 2004; Lynch et al. 2004; Cappuzzo et al. 2005; Bell et al. 2005; Han et al. 2005). This evidence strongly suggests that other mechanisms besides of the *EGFR* mutation status determine the TKI treatment responsiveness (Chang et al. 2011; Cappuzzo et al. 2005; Engelman et al. 2005). Several other predictive biomarkers have also been investigated in relation to NSCLC in order to assess TKI responsiveness. Cappuzzo et al. (2005) reported on *EGFR* amplification and high EGFR protein expression levels associated to gefitinib responsiveness. Likewise, Takano et al. (2005) respective to recurrent NSCLC patients and Hirsch et al. (2006) considering a phase III study of advanced NSCLC subjects determined that an increased *EGFR* gene copy number encompasses a favourable gefitinib sensitivity marker. In addition, significant ErbB-3 over-expression levels have also been associated with gefitinib sensitivity (Engelman et al. 2005).

Furthermore, tumour specimens are required in order to efficiently select patients based on *EGFR* mutation profiles, yet sometimes insufficient primary tumour tissue is available or else circumstances dictate that samples are difficult to obtain having led to *EGFR* gene mutation detection failures (Mitsudomi et al. 2010; Costa et al. 2007).

In this study we have investigated amongst TKI erlotinib treated non-small cell lung cancer (NSCLC) patients the potential predictive outcome of three clinical practice applied serum biomarkers (CEA, CYFRA 21–1, SCC) together with the soluble form of EGFR (sEGFR) and its constituting ligands: epidermal growth factor (EGF), transforming growth factor-alpha (TGF-α) and heparin binding epidermal growth factor (HB-EGF).

## Results

### Patient characteristics

The characteristics of the patients included in this study are displayed in Table 1. The patients’ mean age amounted to 60.81 years (ranging 38–86) and the group consisted mostly of men (67.24%). Never-smokers comprised 24.1% of the patients. Regarding histological types, adenocarcinomas were mainly reported (70.7%) and most tumours were classified into advanced (20.7% stage IIIb) and metastatic (67.2% stage IV) states. Performance status (PS) could only be established for half of the patients: 39.7% encompassed PS 0–1 and 8.6% manifested PS 2–3.

**Table 1:** **Patient and tumour characteristics**

**Variables**	**Demographics**	***N***	**%**
Sex	Male	39	67.2
	Female	19	32.8
Age (ys)	<60	26	44.8
	≥60	32	55.2
Smoking status	Never smoker	14	24.1
	Active	20	34.5
	Ever smoker	24	41.4
Histology	Adenocarcinoma	41	70.7
	Squamous	6	10.3
	Unknown	11	19.0
Stage	I-II	5	8.6
	IIIa	2	3.5
	IIIb	12	20.7
	IV	39	67.2
Performance status	0-1	23	39.7
	2-3	5	8.6
	Unknown	30	51.7
Prior treatment	0	12	20.7
	1	28	48.3
	2	15	25.9
	3	2	3.4
	4	1	1.7

In relation to the previous treatments, patients who had received one previous chemotherapy line (48.3%) had undergone a cisplatin-based doublet chemotherapy pre-treatment. Patients with two different therapies previous to the erlotinib treatment initiation (25.9%) had received cisplatin-based chemotherapy as the first line (except for one patient) and different second line therapies: 7 patients platinum, 5 patients taxane and 3 patients gemcitabine or vinorelbine. A total of three treatments were administered to only 3.4% of the patients: first and second lines consisted of platinum derivatives and with regard to the third line one patient had received gemcitabine while another had been assigned taxane. Only in one case had a patient received more than three treatments whereas the remaining 12 patients had not received any treatment before commencing the erlotinib therapy (Table 1).

### Serum marker concentrations

Table 2 provides the seven serum marker levels providing their average levels together with ranges. The pre-treatment levels of the markers sEGFR, EGF, TGF-α and HB-EGF displayed Gaussian distributions, whilst the levels of CEA, CYFRA 21–1 and SCC entailed skewed distributions.

**Table 2:** **CEA, CYFRA 21–1, SCC, sEGFR, EGF, TGF-α and HB-EGF serum marker concentrations**

**Markers (units)**	**Sample (** ***N*** **)**	**Median**	**Range**
CEA (ng/mL)	35	12.2	0.6 – 10000.0
CYFRA 21–1 (ng/mL)	35	5.5	1.4 – 231.4
SCC (ng/mL)	35	0.9	0.3 – 18.1
sEGFR (ng/mL)	44	59.05	28.4 – 90.2
EGF (pg/mL)	45	757.4	47.4 – 2425.5
TGF-α (pg/mL)	40	18.4	0.01 – 233.2
HB-EGF (pg/mL)	24	157.6	51.3 – 1032.5

### Treatment outcome

The mean erlotinib treatment duration amounted to 6.37 months (spanning from 1–12.0 months). Overall, erlotinib treatment was well tolerated by the majority of the individuals. Following to the erlotinib therapy, clinical responses were assessed giving the following results: no patients exhibited a complete response (CR) to the therapy; the disease control rate covered 47.4%, of which a partial response (PR) was observed in 21.1% of the patients while a stable disease development (SD) was witnessed in 26.3%; whereas disease progression (PD) cases accounted for 52.6% of the patients.

### Survival analysis stratified by patient and tumour characteristics

Patients’ follow-up was performed during a period of approximately 27 months in order to estimate the progression-free survival (PFS) along with the overall survival (OS). During that time period, altogether 51 patients suffered tumour progression of which 48 finally died as a consequence of the lung cancer. The median PFS and OS extents equalled respectively 3.1 (IC 95%: 2.2–4) and 6.97 months (IC 95%: 4.56–9.37). Univariate survival analysis was further carried out in order to assess influences of gender, age, smoking, performance status, histology, tumour stage, previous treatment administration and therapy toxicity on the PFS and OS occurrences of the NSCLC patients (Table 3).

**Table 3:** **Univariate analysis of the clinical and pathological factors in relation to PFS and OS**

			**Progression-free survival**		**Overall survival**		**Univariate cox analysis**					
**Variables**	**Demographics**	***N***	**Me** ^**a**^ **(95% CI)**	***p*** ^***b***^	**Me** ^**a**^ **(95% CI)**	***p*** ^***b***^	**Progression**			**Death**		
							**HR**	**95% CI**	***p***	**HR**	**95% CI**	***p***
Sex	Male	19	2.1(0.8 – 9.1)		10.6(7.0 – 14.3)		1.00			1.00		
	Female	39	0.3(2.2 – 3.4)	0.5	5.4(3.6 – 7.2)	0.383	1.22	0.7 – 2.2	0.501	1.31	0.7 – 2.4	0.385
Age (ys)	<60	26	2.6(2.1 – 3.1)		5.2(3.4 – 7.1)		1.00			1.00		
	≥60	32	4.0(1.8 – 6.1)	0.109	9.7(5.5 – 14)	0.303	0.60	0.4 – 1.1	0.112	0.74	0.4 – 1.3	0.305
Smoking	No smoker	14	6.2(0.0 – 12.9)		10.6(3.4 –17.8)		1.00			1.00		
	Active	44	2.7(2.1 – 3.4)	0.042	5.4(3.6 – 7.2)	0.253	2.00	1.0 – 4.0	0.046	1.5	0.7 – 3.0	0.257
Performance status	0-1	23	2.8(2.2 – 3.5)		6.5(2.6 – 10.4)		1.00			1.00		
	2-3	5	0.4(0.4 – 0.5)	0.254	0.7(0.1 – 1.4)	0.007	1.77	0.66 – 4.8	0.261	3.88	1.3 – 11.1	0.012
Histology	Adenocarcinoma	46	2.8(1.9 – 3.6)		7.7(3.8 – 11.6)		1.00			1.00		
	Squamous	6	2.7(1.5 – 3.9)	0.267	4.4(2.5 – 6.2)	0.045	1.65	0.68 – 4.0	0.273	2.47	0.99 – 6.2	0.054
Stage	I-III	19	2.8(2.2 – 3.5)		10.0(5.4 – 14.6)		1.00			1.00		
	IV	39	3.3(2.0 – 4.6)	0.545	5.8(2.9 – 8.8)	0.315	1.20	0.66 – 2.2	0.546	1.37	0.7 – 2.5	0.318
Prior treatment	No	12	3.1(1.7 – 4.6)		5.0(2.4 – 7.7)		1.00			1.00		
	Yes	46	2.8(2.0 – 3.7)	0.650	7.4(3.5 – 11.3)	0.386	0.86	0.5 – 1.6	0.651	0.74	0.4 – 1.5	0.389
Toxicity	No	7	1.8(1.6 – 2.1)		2.3(1.8 – 2.8)		1.00			1.00		
	Yes	49	3.8(2.2 – 5.3)	<0.001	7.8(4.1 – 11.5)	0.009	0.44	0.21 – 0.93	0.031	0.35	0.15 – 0.8	0.013

*Abbreviations*: *Me* Median, *HR* Hazard Ratio, *CI* Confidence interval.

^a^Months; ^b^p value calculated using the Log-Rank test.

Positive smoking histories presented significantly lower PFS (*p* = 0.042) for active smokers and a hazard ratio (HR) value of 2 of suffering disease progression (*p* = 0.046). Improved performance status at the beginning of the erlotinib treatment (*p* = 0.007) and adenocarcinoma histology (*p* = 0.045) were significantly associated to longer overall survival; however, only a poor performance status remained a significant death predictor as detected by the Cox analysis, exhibiting a 3.88 times higher hazard ratio (*p* = 0.012).

The erlotinib-derived toxicity encompassed a significant prognostic factor with respect to both the progression-free survival (*p* < 0.001) and the overall survival (*p* = 0.009), although with more remarkable differences in the case of the overall survival. Risk assessment confirmed that the toxicity represented a protection factor, with hazard ratios of 0.44 respective to the progression-free survival (*p* = 0.013) and 0.35 regarding the overall survival (*p* = 0.031).

### Patient survival analysis stratified by tumour markers

Table 4 reports the median progression-free survival (PFS) and overall survival (OS) periods of those patients having been classified as holding pre-treatment marker levels below or above of the defined cut-off levels (see Methods), in addition to the results derived from the Cox analysis in order to establish progression and death risks. The patient or sample number disparities compared to Table 2 are due to censored or missing data.

**Table 4:** **Univariate analysis of the serum marker concentrations in relation to PFS and OS**

			**Progression-free survival**		**Overall survival**		**Univariate cox analysis**					
**Markers**	**Levels**	***N***	**Me** ^**a**^ **(95% CI)**	***p*** ^***b***^	**Me** ^**a**^ **(95% CI)**	***p*** ^***b***^	**Progression**			**Death**		
							**HR**	**95% CI**	***p***	**HR**	**95% CI**	***p***
CEA	<5	11	2.8(1.9 – 3.7)		4.4(2.7 – 6.1)		1.00			1.00		
	≥5	24	2.8(1.2 – 4.3)	0.155	10.2(5.9 – 14.5)	<0.001	0.58	0.3 – 1.2	0.161	0.23	0.9 – 0.6	0.001
CYFRA 21-1	<3.3	10	3.2(0.0 – 7.8)		15.0(0.0 – 31.9)		1.00			1.00		
	≥3.3	25	2.8(2.5 – 3.0)	0.317	6.5(4.3 – 8.6)	0.056	1.51	0.67 – 0.38	0.321	2.34	0.95 – 6.0	0.064
SCC	<1.5	29	2.8(2.0 – 3.7)		7.7(3.7 – 11.7)		1.00			1.00		
	≥1.5	6	2.7(2.0 – 3.3)	0.500	6.5(4.7 – 8.3)	0.184	1.36	0.55 – 3.36	0.503	1.87	0.73 – 4.76	0.192
sEGFR	<56.87	20	2.4(1.9 – 2.9)		4.2(0.6 – 7.8)		1.00			1.00		
	≥56.87	24	3.2(0.6 – 5.8)	0.051	9.5(5.3 – 13.6)	0.016	0.53	0.3 – 1.0	0.055	0.43	0.21 – 0.87	0.019
EGF	<713.59	22	3.8(1.9 – 5.6)		7.4(3.2 – 11.6)		1.00			1.00		
	≥713.59	23	2.6(2.0 – 3.2)	0.405	5.1(4.8 – 5.4)	0.488	1.30	0.7 – 2.4	0.408	1.26	0.7 – 2.4	0.490
TGF-α	<21.81	24	2.3(1.5 – 3.1)		5.1(1.5 – 8.7)		1.00			1.00		
	≥21.81	16	2.8(1.8 – 3.7)	0.570	7.7(3.0 – 12.4)	0.732	0.82	0.4 – 1.6	0.572	0.88	0.42 – 1.85	0.733
HB-EGF	<171	14	2.3(0.6 – 4.0)		5.1(1.5 – 8.6)		1.00			1.00		
	≥171	10	3.1(1.5 – 4.7)	0.256	15.0(0.0 – 33.0)	0.093	0.60	0.3 – 1.5	0.262	0.42	0.15 – 1.2	0.104

*Abbreviations*: *Me* Median, *HR* Hazard Ratio, *CI* Confidence interval.

^a^Months; ^b^p value calculated using the Log-Rank test.

Two of the serum markers, namely CEA and sEGFR, were significantly related to an overall survival (OS) prolongation when patients manifested elevated levels. CEA levels above 5 ng/mL had a median OS of 10.2 months, superior to the 4.4 months of patients exhibiting inferior levels (*p* < 0.001). The Cox analysis established a death hazard ratio (HR) of 0.23 with respect to patients with an elevated level of CEA (*p* = 0.001).

As concerned sEGFR, a cut-off value of 56.87 ng/mL clearly differentiated among patients expressing lower levels and experiencing lower median overall survival periods of 4.2 months in contrast to patients displaying levels well above of the cut-off value who presented median overall survival intervals of 9.5 months (*p* = 0.016); progression-free survival differences were nearly significant (*p* = 0.051). The Cox analysis revealed that elevated sEGFR levels implied a diminished death hazard ratio corresponding to 0.43 (*p* = 0.019).

### Relationship between *EGFR* mutation analysis and treatment response

Mutation analysis of the *EGFR* gene TK domain exons 18–21 was carried out on tumour specimens drawn from 33 patients of the study. One patient carried an unspecified mutation type, therefore this individual was further excluded from the subsequent analyses.

*EGFR* gene related mutations were detected in 11 out of the resulting 32 patients (34.4%), while the remaining 21 individuals held the wild type *EGFR* genotype. All the variants detected in this study were located on exons 18, 19 and 21; the most frequent mutation consisted of a deletion in exon 19 (del19) affecting a total of 8 individuals (72.7%), another two cases were detected in exon 21 (L858R and L861Q) and only one mutation case was identified in exon 18 (G719X).

Treatment responsiveness was recorded for 31 patients out of the 32 *EGFR* mutational status appraised cases. Gene mutations were detected in 9 out of 17 (52.9%) cases presenting partial responses (PR) or stable disease (SD) states after undergoing erlotinib administration; however, only 2 mutations were present in the 14 (14.3%) cases of disease progression (PD). Furthermore, *EGFR* mutations were not significantly associated with an improved response (*p* = 0.057, Fisher’s exact Test).

### Patient survival analysis stratified by *EGFR* mutational status

Progression-free survival (PFS) and overall survival (OS) were assessed among the 32 patients characterized by *EGFR* mutations. The median PFS corresponded to 3.47 months (95% CI: 2.59 – 4.34) while the median OS was amounted to 6.53 months (95% CI: 3.99 – 9.07). The Kaplan-Meier analysis and the Log-Rank test detected that the *EGFR* gene mutations conferred significant survival advantages versus the wild type patients, both with respect to the PFS (8.6 versus 2.8 months, *p* = 0.012) and also to the OS (12.6 versus 5.4 months, *p* = 0.033). Univariate hazard ratios of the mutated *EGFR* genotypes corroborated that those patients bearing mutations held minor progression and death probabilities (HR of 0.36 and 0.39, respectively). Results are displayed in Table 5.

**Table 5:** **Univariate analysis of serum markers in patients with** ***EGFR*** **mutation status analysed for survival**

			**Progression-free survival**		**Overall survival**		**Univariate cox analysis**					
**Markers**	**Levels**	***N***	**Me** ^**a**^ **(95% CI)**	***p*** ^***b***^	**Me** ^**a**^ **(95% CI)**	***p*** ^***b***^	**Progression**			**Death**		
							**HR**	**95% CI**	***p***	**HR**	**95% CI**	***p***
*EGFR* mutation		11	8.6 (2.0 – 15.1)		12.6 (4.7 – 21.1)		1.00			1.00		
*Wild type*		21	2.8 (2.0 – 3.6)	0.012	5.4 (4.2 – 6.6)	0.033	0.36	0.16 – 0.82	0.015	0.39	0.16 – 0.95	0.039
CEA	<5	8	3.1 (1.8 – 4.4)		4.2 (2.0 – 6.4)		1.00			1.00		
	≥5	12	2.8 (0.0 – 6.5)	0.237	10.2 (5.0 – 15.0)	0.004	0.55	0.2 – 1.5	0.243	0.21	0.07 – 0.7	0.008
sEGFR	<56.87	20	2.8 (1.7 – 3.8)		4.2 (1.0 – 7.4)		1.00			1.00		
	≥56.87	24	3.8 (0.8 – 6.7)	0.106	9.5 (6.1 – 12.9)	0.013	0.51	0.22 - 1.2	0.112	0.34	0.1 – 0.8	0.016

*Abbreviations*: *Me* Median, *HR* Hazard Ratio, *CI* Confidence interval.

^a^Months; ^b^p value calculated using the Log-Rank test.

### Survival analysis of *EGFR* mutational status characterized patients stratified by tumour markers (sEGFR and CEA)

The tumour markers sEGFR and CEA, which had probed significant survival predictors as identified by the univariate analysis presented in Table 4, were made use of to repeat the survival analysis with respect to the *EGFR* mutational status characterized patients. As had occurred when all of the patients had been analysed, neither the sEGFR nor the CEA tumour markers affected significantly the progression-free survival (PFS). Despite of the statistical power loss, higher sEGFR (*p* = 0.013) and CEA (*p* = 0.004) levels were again associated with a significant overall survival (OS) improvement (Table 5). Survival medians of mutational status characterized patients were practically identical to those that had been obtained for all of the patients assessed in the study. The Cox hazard model also corroborated that these markers encompassed prognostic factors for a lower death risk.

### Patient survival analysis stratified by *EGFR* mutational status combined with CEA and sEGFR marker levels

The mutational *EGFR* status together with the serum CEA and sEGFR marker levels were assayed together in order to quantify a better survival prediction (Table 6). It was possible to only assay pair combinations in relation to survival and risk owing to the low patient number recording simultaneously data with respect to these three variables when all of the three markers bore positive states (mutated *EGFR*, sEGFR and CEA levels above of the cut-off values; 3 patients) or negative (2 patients).

**Table 6:** **Univariate analysis of the combination of** ***EGFR*** **mutational status, CEA and sEGFR for PFS and OS**

			**Progression-free survival**		**Overall survival**		**Univariate cox analysis**					
	**Positive marker** ^**a**^	***N***	**Me** ^**b**^ **(95% CI)**	***p*** ^**c**^	**Me** ^**b**^ **(95% CI)**	***p*** ^**c**^	**Progression**			**Death**		
							**HR**	**95% CI**	***p***	**HR**	**95% CI**	***p***
sEGFR + Mutation Status	2	5	23.5(−)	0.018	9.9(0.0 – 23.0)	0.067	1.000			1.000		
	1	14	7.0(4.9 – 9.0)		2.7(1.8 – 3.7)		2.497	0.782 – 7.979	0.123	4.050	0.904 – 18.148	0.068
	0	9	3.5(0.1 – 6.9)		2.7(1.4 – 4.1)		4.370	1.195 – 15.986	0.026	7.894	1.607 – 38.775	0.011
CEA + Mutation Status	2	4	6.2(0.0 – 13.3)	0.338	6.5(0.6 – 12.5)	0.045	1.000			1.000		
	1	9	2.7(2.3 – 3.1)		10.2(2.8 – 17.6)		2.203	0.648 – 7.717	0.203	1.267	0.324 – 4.953	0.733
	0	7	3.5(2.6 – 4.3)		5.0(1.0 – 9.1)		2.508	0.660 – 9.526	0.177	4.393	1.000 – 19.297	0.050
CEA + sEGFR Patients with known mutation status	2	9	2.7(2.6 – 2.8)	0.689	10.5(9.6 – 11.4)	0.028	1.000			1.000		
	1	8	3.1(0.03 – 6.2)		5.2(3.2 – 7.3)		1.422	0.486 – 4.158	0.520	2.568	0.833 – 7.920	0.101
	0	3	3.5(2.0 – 5.0)		4.2(3.0 – 5.4)		1.787	0.426 – 7.491	0.427	7.433	1.448 – 38.170	0.016
CEA + sEGFR all patients	2	14	3.2(0.0 – 7.2)	0.229	12.6(5.7 – 19.4)	0.003	1.000			1.000		
	1	16	2.6(1.9 – 3.4)		5.2(2.4 – 8.0)		1.931	0.840 – 7.305	0.121	2.962	1.170 – 7.496	0.022
	0	4	2.5(1.4 – 3.7)		3.5(1.6 – 5.3)		2.192	0.658 – 7.305	0.201	8.359	2.111 – 33.095	0.002

*Abbreviations*: *Me* Median, *HR* Hazard Ratio, *CI* Confidence interval.

^a^(2) both markers positive (above the cut-off point or mutated EGFR), (1) only one positive and (0) both negative (below the cut-off point or WT EGFR); ^b^Months; ^c^p value calculated using the Log-Rank test.

Only the *EGFR* mutational status and the sEGFR level associated together significantly with a longer progression-free survival (PFS) embracing a median of 23.5 months when the *EGFR* gene was mutated and the protein serum levels displayed increased values, compared to the 3.5 months in the case of patients with the non-mutated gene also exhibiting inferior sEGFR levels (*p* = 0.018) altogether enclosing a risk of progression of 4.37 (*p* = 0.026). Conversely, the *EGFR* mutation type did not maintain a high significance with respect to the PFS when combined with the CEA marker level, nor did the combination of the high sEGFR and CEA levels affect favourably the PFS.

On the contrary, concerning all three combinations taken together better survival times were exhibited when at least one of the markers had elevated levels or else the *EGFR* gene was mutated, although in the case of the sEGFR level and the *EGFR* gene mutational status the significance threshold was not reached. The univariate Cox analysis in relation to the overall-survival (OS) revealed similar propensity-death hazard ratios in the case of low sEGFR levels when combined with either the non-mutated *EGFR* genotype or low CEA levels, 7.89 (*p* = 0.011) and 7.43 (*p* = 0.016) respectively. The combination of the low CEA levels together with the non-mutated *EGFR* status implied an increased death risk of 4.39 (*p* = 0.050).

**Figure 1:**
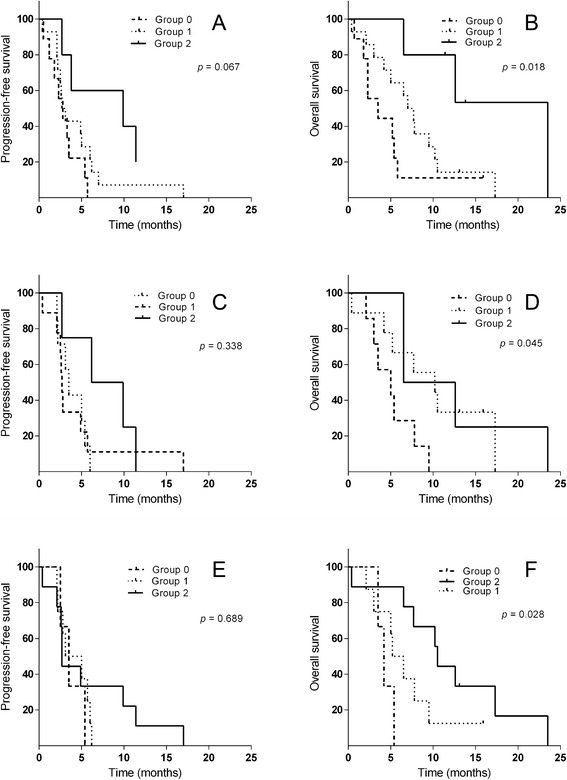
PFS and OS according to combination of sEGFR and CEA concentrations and EGFR mutational status. Groups consisted of the following: (0) patients with sEGFR levels < 56.87 ng/mL or CEA < 5 ng/mL and a negative *EGFR* gene mutational status, (1) only one positive marker, (2) patients with sEGFR > 56.87 ng/mL or CEA > 5 ng/mL and a positive *EGFR* gene mutational status. (**A**) and (**B**) Kaplan-Meier curves respectively of PFS and OS according to the combination of the sEGFR serum marker levels and the *EGFR* gene mutational status. PFS (**C**) and OS (**D**) curves in relation to the combination of the CEA serum levels and the *EGFR* gene mutational status. PFS (**E**) and OS (**F**) curves corresponding to the combination of the CEA and sEGFR serum marker levels.

Figure 1 presents according to the combination of the sEGFR and CEA serum marker levels in conjunction with the *EGFR* mutational gene status the Kaplan Meier curves of the progression-free survival (PFS) and of the overall survival (OS).

### Progression-free survival and overall survival multivariate analysis

A multivariate Cox analysis was performed in order to assess which variables would be independent survival predictors of erlotinib treated NSCLC patients (Table 7). Data regarding Performance status (PS) and CEA serum marker levels were excluded from the multivariate model, thus remaining a total of 25 patients for further analysis.

**Table 7:** **Multivariate models corresponding to progression-free survival and overall survival**

	**Progression-free survival**			**Overall survival**		
	**HR**	**95% CI**	***p*** ^**a**^	**HR**	**95% CI**	***p*** ^**a**^
sEGFR						
<56.87	1.000			1.000		
≥56.87	0.406	0.151 – 1.089	0.073	0.271	0.096 – 0.760	0.013
Mutational status						
Wild type	1.000			1.000		
*EGFR* Mutated	0.716	0.218 – 2.354	0.583	0.726	0.196 – 2.696	0.633
Toxicity						
No	1.000			1.000		
Yes	0.201	0.047 – 0.854	0.030	0.088	0.018 – 0.425	0.002

*Abbreviations*: *HR* Hazard Ratio, *CI* Confidence interval.

^a^Wald test is used to calculate HR.

Patients with mutated *EGFR* genotypes, sEGFR serum marker levels above of the cut-off value of 56.87 ng/mL and displaying toxicity symptoms presented diminished progression and death risks compared to the patients who exhibited the opposed characteristics. Nevertheless, only sEGFR serum marker levels and erlotinib toxicity resulted significant independent predictors of overall survival (OS), whereas only the erlotinib toxicity was also significant in the case of the progression-free survival (PFS).

The death risk of patients with elevated sEGFR serum marker levels comprised 0.27 (*p* = 0.013). Furthermore, patients suffering from erlotinib toxicity exhibited a much better prognosis, with poor progression (HR = 0.201, *p* = 0.030) and death (HR = 0.088, *p* = 0.002) risks.

## Discussion

Serum biomarkers to predict the survival of NSCLC patients treated with erlotinib (Tarceva®) were investigated in this study. Dating back to the development of the EURTAC (Rosell et al. 2012) and OPTIMAL studies (Zhou et al. 2011), the *EGFR* gene mutation status has been widely applied to select patients most likely to respond to the EGFR-TKI treatments. Nonetheless, it needs to be indicated that not all patients carrying *EGFR* gene mutations respond receptively to EGFR-TKI treatments (Mitsudomi et al. 2010; Tamura et al. 2008; Maemondo et al. 2010) and also, on the other hand, that no mutations are identified in 10-20% of patients with partial responses to EGFR-TKIs (Pao et al. 2004; Lynch et al. 2004; Cappuzzo et al. 2005; Bell et al. 2005; Han et al. 2005). Therefore, the quest of different sensitivity prediction methods to TKI based therapy in lung cancer remains an issue of interest.

In this study we have tested in pre-treatment serum several markers − such as sEGFR together with its associated ligands TGF-α, EGF and HB-EGF − in relationship to survival of NSCLC patients treated with the EGFR-TKI erlotinib. Routine clinical markers were also assessed: CEA, CYFRA 21–1 and SCC.

Globally, our median overall survival-OS and progression free-survival-PFS outcomes are coincident to those described in the main erlotinib drug evaluation studies (Shepherd et al. 2005). The Kaplan-Meier analysis revealed that only the performance status-PS, tumour histology and toxicity had a substantial impact on the OS of the erlotinib treated patients. The univariate analysis corroborated that PS 2–3 in addition to no toxicity development were significantly associated with a poor prognosis. In coincidence with other previous studies (Petrelli et al. 2012; Emery et al. 2009), toxicity has represented a protection factor indicative of a good clinical response. In the case of adenocarcinoma histology, our results are also in line with those of preceding works (Tsao et al. 2011; Jung et al. 2012).

In our study patients with pre-treatment sEGFR levels higher than 56.87 ng/mL evidenced longer progression free-survival (PFS) while especially overall survival (OS) phases compared to patients containing lower serum levels. Previously, Gregorc et al. (2004) and also Kappers et al. (2010) had already obtained similar results observing that patients embracing higher pre-treatment sEGFR serum levels were more likely to respond receptively. Nonetheless, other authors have described the opposite scenario disclosing discrepant sEGFR levels compared to most of the information available (Kasahara et al. 2010; Lemos-González et al. 2007), probably owing to the different methodology employed.

Of the different EGFR-specific ligands tested, none showed a significant association with patient survival. In the case of the serum marker HB-EGF a cut-off value equal to 171.07 pg/mL separated those patients comprising higher levels while presenting more extended progression free-survival-PFS and overall survival-OS episodes, although statistical significance was not reached. These results are in direct contradiction to those of other authors who have described high TGF-α and HB-EGF levels to be associated with a progressive disease and a shorter overall survival-OS lapse after NSCLC patients had been treated with gefitinib (Masago et al. 2008).

Among the diverse clinical markers analysed in our study, only the serum marker CEA revealed a statistical significant relation with the treated patients’ survival. Our finding establishing that high CEA levels constituted good predictors of survival regardless of the histology was a highly surprising and unexpected fact, as previously in other studies regarding patients with more advanced cancer stages who had been receiving chemotherapy these had displayed high CEA levels which had been associated with a poor prognosis (Lin et al. 2012). Nevertheless, other authors have made similar observations to ours in advanced NSCLC patients undergoing TKI based treatments (Jung et al. 2012; Okamoto et al. 2005). In the first study (Jung et al. 2012), high pre-treatment CEA levels were significantly associated with longer progression-free survival interludes. Similarly, Okamoto et al. (2005) reported that EGFR TKI treated patients with high pre-treatment CEA levels bestowed longer survival terms and displayed better responses than those patients encompassing lower CEA levels.

In relation to the serum marker CYFRA 21–1, levels above of the cut-off limit showed lower overall survival-OS rates, although the statistical significance threshold was not surpassed. These results are very similar to those described by Chen et al. (2010), who determined that this marker’s pre-treatment levels might provide prognostic information in the case of gefitinib treatments.

So far few reports exist regarding the relationship between serological marker levels and the curative effect of erlotinib. The associated shortcomings of these studies include an *EGFR* mutation test lack, the very low patient number (Ishikawa et al. 2005), or else only wild-type patient analysis (Chang et al. 2011).

We have included in our study data pertaining to the *EGFR* mutational status of 32 characterised patients (tumour tissue was not available for the remaining subjects). Mutations were detected among 11 patients with a resulting *EGFR* gene mutation rate of 34%. This percentage is markedly superior to the *EGFR* gene mutation frequency reported for European Caucasians covering 9.8% in the case of German NSCLC patients (Gahr et al. 2013) and amounting to 16.6% with respect to a Spanish population of advanced NSCLC patients (Rosell et al. 2009). The *EGFR* gene mutation frequency discrepancies are best explained on the basis of the selection criteria employed in this study restricting the eligible NSCLC patients to those receiving an erlotinib treatment, in spite of including all of the NSCLC patients, together with some patient’s non-availability of tumour tissue which precluded an accurate *EGFR* gene mutation estimate.

Our results related to the *EGFR* mutational status indicated a strong association (Fisher analysis close to significance) between the *EGFR* mutations present and the response to the erlotinib treatment. Furthermore, the mutational status holds a prognostic value as the mutated *EGFR* gene patients displayed higher overall-survival (OS) and progression free-survival (PFS) episodes in comparison to patients lacking mutations, also corroborated by the univariate Cox analysis which revealed that progression and death are less prone to occur in patients bearing mutations. Our data agrees with that of previous studies (Inoue et al. 2006; Rosell et al. 2012; Tamura et al. 2008; Sequist et al. 2008).

As was also observed in the case of Chang et al. (2011), some patients responded positively to erlotinib medication although they presented the wild type *EGFR* genotype: 4.8% manifested partial responses (PR), 57.1% exhibited stable disease development (SD) while 33.3% incurred in disease progression (PD).

Death predictive markers disclosed by the univariate analysis, the *EGFR* gene mutational status in addition to the serum CEA and sEGFR marker levels, were further assayed in combination in order to assess better survival predictions. Marker combinations were evaluated among three categories: (2) both positive markers (above of the cut-off threshold or else a mutated *EGFR* genotype), (1) only one marker positive and (0) both markers negative (below of the cut-off threshold or else a wild type *EGFR* genotype). The combination of the sEGFR level together with the *EGFR* gene mutational status did not have a significant impact on the overall survival (OS) as revealed by the Kaplan-Meier analysis, even though the median survival time was the highest for elevated sEGFR levels and mutated *EGFR* genes, while the Cox analysis indicated that possessing wild type *EGFR* tumours together with low receptor levels present in the pre-treatment serum entailed a death risk factor, holding those patients a hazard ratio (HR) 7.89 times higher in contrast to patients with tumours bearing a mutated *EGFR* gene and expressing high sEGFR levels. Accordingly, we do not consider the sEGFR level in conjunction with the *EGFR* gene mutational status a worthy combination.

Noticeably, sEGFR and CEA levels combined together significantly determined overall survival (OS), thus having represented a more useful combination that achieves longer survival in erlotinib-treated patients. Combined high pre-treatment serum CEA and sEGFR levels clearly indicated a better prognosis granted that these patients showed higher OS (12.6 months) time frames weighed against those individuals displaying only one marker with an elevated level (5.2 months), whilst particularly in contrast to those patients with both markers expressing low levels (3.5 months), all confirmed by the Cox analysis disclosing that group 0 patients sustained a hazard ratio (HR) 8.36 times higher than that of the group 2 patients. Almost the same results could be observed when only the patients with a known mutational status were considered. Compared to the single *EGFR* mutational status determination, it remains worthy to highlight that the combined CEA and sEGFR use allowed to discern 14 patients (41%) with a prolonged OS, whereas the mutational status analysis permitted to distinguish 11 patients (34.3%); moreover, the OS benefit of both the positive sEGFR and CEA levels combined was at least as good as the survival increase of patients with the mutated *EGFR* gene, in both cases spanning 12.6 months. Nonetheless, it should be kept in mind that the prognostic value incremental evaluation of the sEGFR and CEA levels combined to that of the *EGFR* gene mutational status standing alone was not performed due to insufficient available data, as has already been explained elsewhere. The before mentioned results suggest that the combined CEA and sEGFR usage may provide an equivalent prognostic information to that of the *EGFR* gene mutational status with regard to determining NSCLC erlotinib treated patient prognosis.

Using a multivariate Cox regression model only the pre-treatment sEGFR level together with erlotinib toxicity remained significant survival predictors. Although all of the variables with a significant death risk impact should have been included in the univariate analysis: performance status (PS), erlotinib toxicity, *EGFR* gene mutational status and pre-treatment serum levels of the sEGFR and CEA markers, we could not include in the multivariate analysis the marker CEA or the performance status due to an insufficient amount of data. Reinforcing our results, Gregorc et al. (2004) had previously studied the sEGFR levels of patients with advanced NSCLC stages observing that high sEGFR levels were significant with respect to the three multivariate analyses performed.

Our study presents some weaknesses; in this line our main concern contemplates the limited number of recruited individuals owing to the peculiarities of the selected subjects, in addition to an insufficient serum volume, which precluded the whole population determination of all of the markers. In the future, larger studies to corroborate the preliminary prognosis conclusions of the erlotinib-treated patients based on combined sEGFR and CEA data are needed that, in addition, could also confirm the potential of other biomarkers that might have been underestimated, such as could be the case of the HB-EGF and CYFRA 21.1 serum markers, whose levels showed survival and risk relations close to significance. Despite of these shortcomings, the high CEA and sEGFR prognostic value similarity displayed for the whole population survival analysis as compared to the subgroup analyzed for the *EGFR* gene mutational status indicates that the limited sample size has not affected the results.

Secondly, as our patient cohort was heterogeneous in relation to the previous treatment, caution should be exercised in drawing any firm conclusions. Our analyses have all been based on erlotinib-treated patient outcomes, given that our study lacked a control group (patients who had not received erlotinib treatment), thus, results do not allow to differentiate whether the survival benefit was due to a prognostic or predictive value of the serum markers sEGFR and CEA to identify patients who would respond positively to erlotinib therapy (Coate et al. 2009).

## Conclusions

The present study has indicated that pre-treatment levels above 5 ng/mL of CEA or above 56.87 ng/mL of sEGFR comprise survival markers designed for NSCLC patients treated with erlotinib. The combined assessment of sEGFR and CEA serum marker levels could be of value in order to pre-select patients to undergo EGFR-TKI treatments. This is in particular relevant in circumstances when tumour tissues are insufficient or else the hospital lacks the appropriate facilities, in view of the fact that sEGFR and CEA level determinations are routinely feasible whilst relatively non-invasive and inexpensive procedures.

## Methods

### Patients and treatment assessment response

A total of 58 patients were included in this study diagnosed with NSCLC who had been treated with the TKI erlotinib at the Complejo Hospitalario Universitario de Vigo (Spain) from July 2009 to June 2011. The inclusion criterion comprised all patients who had received erlotinib medication during the study period as first or second-line treatment, these patients had failed to respond to conventional intravenous chemotherapy or had been unable to receive chemotherapy. Patients were treated with daily doses of 150 mg and dose reductions of 50 mg were undertaken in cases of observing unacceptable toxicity.

Performance status (PS) and the treatments received prior to the erlotinib therapy were recorded. PS refers to the manner in which the patient’s disease progresses affecting the daily vital capabilities, thus being applied to determine an appropriate treatment and prognosis. PS data was collected following the Eastern Cooperative Oncology Group scale (Oken et al. 1982). Erlotinib-derived toxicity data was also collected denoting any toxicity type experienced by patients due to the treatment received.

The objective tumour response was assessed every 3 months after the beginning of the treatment by means of a computerized tomography according to the Response Evaluation Criteria of Solid Tumors (Eisenhauer et al. 2009). Each patient’s response was then classified into one of the following categories: responders, including cases of complete response (CR), partial response (PR) and stable disease development (SD); and non responders, including cases of disease progression (PD).

All of the patients provided an informed consent before of the enrolment in the study and marker determination was accomplished without interrupting the normal clinical practices. The study followed all of the guidelines set up to undertake experimental investigation as required by the authors’ institutions and complied fully with the Helsinki Declaration.

### Serum marker level determinations

Blood samples were collected before of the EGFR-TKI treatment initiation. Separated serums were stored at −25°C until further use.

CEA and CYFRA 21–1 measurements were carried out by electrochemiluminescence immunoassays (ECLIA, Roche Diagnostics, Germany) using an Elecsys® 2010 analyzer. The SCC antigen determination was accomplished by means of the TRACE technology (Time-Resolved Amplified Cryptate Emission) making use of a KRIPTOR BRAHMS-ATOM® apparatus.

The *DuoSet® ELISA Development System human (*R&D *Systems* Minneapolis, MN) kits were used in order to determine the sEGFR, EGF, HB-EGF and TGF-*α* concentrations present in the serums, performed according to manufacturer’s instructions. Optical densities were read by way of an EnVision Multilabel Plate Reader (Perkin Elmer). Final sample sizes to determine marker concentrations depended on the patient’s available serum quantities.

### *EGFR* gene mutation analysis

Tumour tissue samples for *EGFR* mutation testing were available only for 33 patients (12 women, 20 men, one patient was excluded due to an unspecified mutation type). The Cobas *EGFR* Mutation Test kit (Roche Molecular Systems, Inc., Branchburg, USA) entailing a CE-IVD-marked allele-specific PCR test designed to detect the presence of 41 mutations within exons 18, 19, 20 and 21 of the TK domain was applied to paraffin-embedded tissue blocks. Data analysis and interpretation was implemented through the computer software Cobas z 480 (Roche Molecular Systems, Inc., Branchburg, USA).

### Statistical analysis

Normality of the continuous variable distributions was assessed using the Kolmogorov-Smirnov test. Variable distribution differences between and among groups regarding the *EGFR* mutational status were compared using Fisher’s exact test. Receiver operating characteristic curves (ROC) were drawn for the novel tumour markers (sEGFR, EGF, HB-EGF and TGF-α) in order to assess their discerning value to tell apart the non-progressive from progressive patients and also to establish the survival analysis cut-off values: 56.87 ng/mL in the case of sEGFR, 713.59 pg/mL with respect to EGF, 171.07 pg/mL regarding HB-EGF and 21.81 pg/mL concerning TGF-α. The pertinent cut-off values of CEA, CYFRA 21–1 and SCC were respectively established at 5 ng/mL, 3.3 ng/mL and 1.5 ng/mL.

The progression-free survival (PFS), the overall survival (OS) and the 95% confidence intervals (95% CI) were assessed through the Kaplan-Meier method while survival differences between patients’ groups were compared by means of the Log-Rank test. PFS values were calculated starting the date of erlotinib treatment initiation up to the date of the first PD appearance or else up to the date of the last contact. The OS values were estimated from the date of therapy initiation up to the death date arising from any cause or else up to the date of the last contact. The association of risk factors with survival was evaluated according to the Cox proportional hazards regression model. The multivariate analysis was performed using a logistic regression model in order to identify those variables associating independently with survival. Statistical significances were defined at the *p* < 0.05 probability level. The statistical analysis was carried out using the software package SPSS V15 run under Windows (Copyright© SPSS Inc. 1989–2002, Chicago, IL).

## Abbreviations

CI: Confidence interval
EGF: Epidermal growth factor
EGFR: Epidermal growth factor receptor
HB-EGF: Heparin binding epidermal growth factor
TKI: Tyrosine kinase inhibitors
Me: Median
NSCLC: Non-small cell lung cancer
OS: Overall survival
PFS: Progression-free survival
TGF-α: Transforming growth factor-alpha
WT: Wild type

## References

[CR1] Baselga J (2002). Why the epidermal growth factor receptor? The rationale for cancer therapy. Oncologist.

[CR2] Bell DW, Lynch TJ, Haserlat SM, Harris PL, Okimoto RA, Brannigan BW, Sgroi DC, Muir B, Riemenschneider MJ, Iacona RB, Krebs AD, Johnson DH, Giaccone G, Herbst RS, Manegold C, Fukuoka M, Kris MG, Baselga J, Ochs JS, Haber DA (2005). Epidermal growth factor receptor mutations and gene amplification in non-small-cell lung cancer: molecular analysis of the IDEAL/INTACT gefitinib trials. J Clin Oncol.

[CR3] Cappuzzo F, Hirsch FR, Rossi E, Bartolini S, Ceresoli GL, Bemis L, Haney J, Witta S, Danenberg K, Domenichini I, Ludovini V, Magrini E, Gregorc V, Doglioni C, Sidoni A, Tonato M, Franklin WA, Crino L, Bunn PA, Varella-Garcia M (2005). Epidermal growth factor receptor gene and protein and gefitinib sensitivity in non-small-cell lung cancer. J Natl Cancer Inst.

[CR4] Cappuzzo F, Ligorio C, Jänne PA, Toschi L, Rossi E, Trisolini R, Paioli D, Holmes AJ, Magrini E, Finocchiaro G, Bartolini S, Cancellieri A, Ciardiello F, Patelli M, Crino L, Varella-Garcia M (2007). Prospective study of gefitinib in epidermal growth factor receptor fluorescence in situ hybridization-positive/phospho-Akt-positive or never smoker patients with advanced non-small-cell lung cancer: the ONCOBELL trial. J Clin Oncol.

[CR5] Chang MH, Ahn HK, Lee J, Jung CK, Choi YL, Park YH, Ahn JS, Park K, Ahn MJ (2011). Clinical impact of amphiregulin expression in patients with epidermal growth factor receptor (EGFR) wild-type nonsmall cell lung cancer treated with EGFR-tyrosine kinase inhibitors. Cancer.

[CR6] Chen F, Luo X, Zhang J, Lu Y, Luo R (2010). Elevated serum levels of TPS and CYFRA 21–1 predict poor prognosis in advanced non-small-cell lung cancer patients treated with gefitinib. Med Oncol.

[CR7] Ciardiello F, Tortora G (2008). EGFR antagonists in cancer treatment. N Engl J Med.

[CR8] Coate LE, John T, Tsao MS, Shepherd FA (2009). Molecular predictive and prognostic markers in non-small-cell lung cancer. Lancet Oncol.

[CR9] Costa DB, Kobayashi S, Tenen DG, Huberman MS (2007). Pooled analysis of the prospective trials of gefitinib monotherapy for EGFR-mutant non-small cell lung cancers. Lung Cancer.

[CR10] Eisenhauer EA, Therasse P, Bogaerts J, Schwartz LH, Sargent D, Ford R, Dancey J, Arbuck S, Gwyther S, Mooney M, Rubinstein L, Shankar L, Dodd L, Kaplan R, Lacombe D, Verweij J (2009). New response evaluation criteria in solid TUMORs: revised RECIST guideline (version 1.1). Eur J Cancer.

[CR11] Emery IF, Battelli C, Auclair PL, Carrier K, Hayes DM (2009). Response to gefitinib and erlotinib in Non-small cell lung cancer: a restrospective study. BMC Cancer.

[CR12] Engelman JA, Jänne PA, Mermel C, Pearlberg J, Mukohara T, Fleet C, Cichowski K, Johnson BE, Cantley LC (2005). ErbB-3 mediates phosphoinositide 3-kinase activity in gefitinib-sensitive non-small cell lung cancer cell lines. Proc Natl Acad Sci USA.

[CR13] Gahr S, Stoehr R, Geissinger E, Ficker JH, Brueckl WM, Gschwendtner A, Gattenloehner S, Fuchs FS, Schulz C, Rieker RJ, Hartmann A, Ruemmele P, Dietmaier W (2013). EGFR mutational status in a large series of Caucasian European NSCLC patients: data from daily practice. Br J Cancer.

[CR14] Gregorc V, Ceresoli GL, Floriani I, Spreafico A, Bencardino KB, Ludovini V, Pistola L, Mihaylova Z, Tofanetti FR, Ferraldeschi M, Torri V, Cappuzzo F, Crinò L, Tonato M, Villa E (2004). Effects of gefitinib on serum epidermal growth factor receptor and HER2 in patients with advanced non-small cell lung cancer. Clin Cancer Res.

[CR15] Han SW, Kim TY, Hwang PG, Jeong S, Kim J, Choi IS, Oh DY, Kim JH, Kim DW, Chung DH, Im SA, Kim YT, Lee JS, Heo DS, Bang YJ, Kim NK (2005). Predictive and prognostic impact of epidermal growth factor receptor mutation in non-small-cell lung cancer patients treated with gefitinib. J Clin Oncol.

[CR16] Hirsch FR, Varella-Garcia M, Bunn PA, Franklin WA, Dziadziuszko R, Thatcher N, Chang A, Parikh P, Pereira JR, Ciuleanu T, von Pawel J, Watkins C, Flannery A, Ellison G, Donald E, Knight L, Parums D, Botwood N, Holloway B (2006). Molecular predictors of outcome with gefitinib in a phase III placebo-controlled study in advanced non-small-cell lung cancer. J Clin Oncol.

[CR17] Inoue A, Suzuki T, Fukuhara T, Maemondo M, Kimura Y, Morikawa N, Watanabe H, Saijo Y, Nukiwa T (2006). Prospective phase II study of gefitinib for chemotherapy-naive patients with advanced non-small-cell lung cancer with epidermal growth factor receptor gene mutations. J Clin Oncol.

[CR18] Iressa® (2005) (gefitinib) tablets, oral [package insert] AstraZeneca Pharmaceuticals.

[CR19] Ishikawa N, Daigo Y, Takano A, Taniwaki M, Kato T, Hayama S, Murakami H, Takeshima Y, Inai K, Nishimura H, Tsuchiya E, Kohno N, Nakamura Y (2005). Increases of amphiregulin and transforming growth factor-alpha in serum as predictors of poor response to gefitinib among patients with advanced non-small cell lung cancers. Cancer Res.

[CR20] Janne PA, Engelman JA, Johnson BE (2005). Epidermal growth factor receptor mutations in non-small-cell lung cancer: implications for treatment and tumor biology. J Clin Oncol.

[CR21] Jemal A, Bray F, Center MM, Ferlay J, Ward E, Forman D (2010). Global cancer statistics. CA Cancer J Clin.

[CR22] Jung M, Kim SH, Hong S, Kang YA, Kim SK, Chang J, Rha SY, Kim JH, Kim DJ, Cho BC (2012). Prognostic and predictive value of carcinoembryonic antigen and cytokeratin-19 fragments levels in advanced non-small cell lung cancer patients treated with gefitinib or erlotinib. Yonsei Med J.

[CR23] Kappers I, Vollebergh MA, van Tinteren H, Korse CM, Nieuwenhuis LL, Bonfrer JM, Klomp HM, van Zandwijk N, van den Heuvel MM (2010). Soluble Epidermal Growth Factor Receptor (sEGFR) and Carcinoembryonic Antigen (CEA) concentration in patients with non-small cell lung cancer: correlation with survival after erlotinib and gefitinib treatment. Ecancermedicalscience.

[CR24] Kasahara K, Arao T, Sakai K, Matsumoto K, Sakai A, Kimura H, Sone T, Horiike A, Nishio M, Ohira T, Ikeda N, Yamanaka T, Saijo N, Nishio K (2010). Impact of serum hepatocyte growth factor on treatment response to epidermal growth factor receptor tyrosine kinase inhibitors in patients with non-small cell lung adenocarcinoma. Clin Cancer Res.

[CR25] Kim ES, Hirsh V, Mok T, Socinski MA, Gervais R, Wu YL, Li LY, Watkins CL, Sellers MV, Lowe ES, Sun Y, Liao ML, Osterlind K, Reck M, Armour AA, Shepherd FA, Lippman SM, Douillard JY (2008). Gefitinib versus docetaxel in previously treated non-small-cell lung cancer (INTEREST): a randomised phase III trial. Lancet.

[CR26] Lemos-González Y, Rodríguez-Berrocal FJ, Cordero OJ, Gómez C, Páez de la Cadena M (2007). Alteration of the serum levels of the epidermal growth factor receptor and its ligands in patients with non-small cell lung cancer and head and neck carcinoma. Br J Cancer.

[CR27] Lin XF, Wang XD, Sun DQ, Li Z, Bai Y (2012). High serum CEA and CYFRA21-1 levels after a Two-cycle adjuvant chemotherapy for NSCLC: possible poor prognostic factors. Cancer Biol Med.

[CR28] Lynch TJ, Bell DW, Sordella R, Gurubhagavatula S, Okimoto RA, Brannigan BW, Harris PL, Haserlat SM, Supko JG, Haluska FG, Louis DN, Christiani DC, Settleman J, Haber DA (2004). Activating mutations in the epidermal growth factor receptor underlying responsiveness of non-small-cell lung cancer to gefitinib. N Engl J Med.

[CR29] Maemondo M, Inoue A, Kobayashi K, Sugawara S, Oizumi S, Isobe H, Gemma A, Harada M, Yoshizawa H, Kinoshita I, Fujita Y, Okinaga S, Hirano H, Yoshimori K, Harada T, Ogura T, Ando M, Miyazawa H, Tanaka T, Saijo Y, Hagiwara K, Morita S, Nukiwa T (2010). Gefitinib or chemotherapy for non-small-cell lung cancer with mutated EGFR. N Engl J Med.

[CR30] Masago K, Fujita S, Hatachi Y, Sugawara S, Oizumi S, Isobe H, Gemma A, Harada M, Yoshizawa H, Kinoshita I, Fujita Y, Okinaga S, Hirano H, Yoshimori K, Harada T, Ogura T, Ando M, Miyazawa H, Tanaka T, Saijo Y, Hagiwara K, Morita S, Nukiwa T, North-East Japan Study Group (2008). Clinical significance of pretreatment serum amphiregulin and transforming growth factor-alpha, and an epidermal growth factor receptor somatic mutation in patients with advanced non-squamous, non-small cell lung cancer. Cancer Sci.

[CR31] Mitsudomi T, Morita S, Yatabe Y, Negoro S, Okamoto I, Tsurutani J, Seto T, Satouchi M, Tada H, Hirashima T, Asami K, Katakami N, Takada M, Yoshioka H, Shibata K, Kudoh S, Shimizu E, Saito H, Toyooka S, Nakagawa K, Fukuoka M, West Japan Oncology Group (2010). Gefitinib versus cisplatin plus docetaxel in patients with non-small-cell lung cancer harbouring mutations of the epidermal growth factor receptor (WJTOG3405): an open label, randomised phase 3 trial. Lancet Oncol.

[CR32] Okamoto T, Nakamura T, Ikeda J, Maruyama R, Shoji F, Miyake T, Wataya H, Ichinose Y (2005). Serum carcinoembryonic antigen as a predictive marker for sensitivity to gefitinib in advanced non-small cell lung cancer. Eur J Cancer.

[CR33] Oken MM, Creech RH, Tormey DC, Horton J, Davis TE, McFadden ET, Carbone PP (1982). Toxicity and response criteria of the Eastern Cooperative Oncology Group. Am J Clin Oncol.

[CR34] Pao W, Miller V, Zakowski M, Doherty J, Politi K, Sarkaria I, Singh B, Heelan R, Rusch V, Fulton L, Mardis E, Kupfer D, Wilson R, Kris M, Varmus H (2004). EGF receptor gene mutations are common in lung cancers from “never smokers” and are associated with sensitivity of tumors to gefitinib and erlotinib. Proc Natl Acad Sci USA.

[CR35] Petrelli F, Borgonovo K, Cabiddu M, Lonati V, Barni S (2012). Relationship between skin rash and outcome in non-small-cell lung cancer patients treated with anti-EGFR tyrosine kinase inhibitors: a literature-based meta-nalysis of 24 trials. Lung Cancer.

[CR36] Rosell R, Moran T, Queralt C, Porta R, Cardenal F, Camps C, Majem M, Lopez-Vivanco G, Isla D, Provencio M, Insa A, Massuti B, Gonzalez-Larriba JL, Paz-Ares L, Bover I, Garcia-Campelo R, Moreno MA, Catot S, Rolfo C, Reguart N, Palmero R, Sánchez JM, Bastus R, Mayo C, Bertran-Alamillo J, Molina MA, Sanchez JJ, Taron M, Spanish Lung Cancer Group (2009). Screening for epidermal growth factor receptor mutations in lung cancer. N Engl J Med.

[CR37] Rosell R, Carcereny E, Gervais R, Vergnenegre A, Massuti B, Felip E, Palmero R, Garcia-Gomez R, Pallares C, Sanchez JM, Porta R, Cobo M, Garrido P, Longo F, Moran T, Insa A, De Marinis F, Corre R, Bover I, Illiano A, Dansin E, de Castro J, Milella M, Reguart N, Altavilla G, Jimenez U, Provencio M, Moreno MA, Terrasa J, Muñoz-Langa J (2012). Erlotinib versus standard chemotherapy as first-line treatment for European patients with advanced EGFR mutation-positive non-small-cell lung cancer (EURTAC): a multicentre, open-label, randomised phase 3 trial. Lancet Oncol.

[CR38] Sequist LV, Martins RG, Spigel D, Grunberg SM, Spira A, Jänne PA, Joshi VA, McCollum D, Evans TL, Muzikansky A, Kuhlmann GL, Han M, Goldberg JS, Settleman J, Iafrate AJ, Engelman JA, Haber DA, Johnson BE, Lynch TJ (2008). First-line gefitinib in patients with advanced non-small-cell lung cancer harboring somatic EGFR mutations. J Clin Oncol.

[CR39] Shepherd FA, Rodrigues Pereira J, Ciuleanu T, Tan EH, Hirsh V, Thongprasert S, Campos D, Maoleekoonpiroj S, Smylie M, Martins R, van Kooten M, Dediu M, Findlay B, Tu D, Johnston D, Bezjak A, Clark G, Santabárbara P, Seymour L, National Cancer Institute of Canada Clinical Trials Group (2005). National Cancer Institute of Canada Clinical Trials Group. Erlotinib in previously treated non-small cell lung cancer. N Engl J Med.

[CR40] Takano T, Ohe Y, Sakamoto H, Tsuta K, Matsuno Y, Tateishi U, Yamamoto S, Nokihara H, Yamamoto N, Sekine I, Kunitoh H, Shibata T, Sakiyama T, Yoshida T, Tamura T (2005). Epidermal growth factor receptor gene mutations and increased copy numbers predict gefitinib sensitivity in patients with recurrent non-small-cell lung cancer. J Clin Oncol.

[CR41] Tamura K, Okamoto I, Kashii T, Negoro S, Hirashima T, Kudoh S, Ichinose Y, Ebi N, Shibata K, Nishimura T, Katakami N, Sawa T, Shimizu E, Fukuoka J, Satoh T, Fukuoka M, West Japan Thoracic Oncology Group (2008). Multicentre prospective phase II trial of gefitinib for advancednon-small cell lung cancer with epidermal growth factor receptor mutations: results of the West Japan Thoracic Oncology Group trial (WJTOG0403). Br J Cancer.

[CR42] Tarceva® (2009). (erlotinib) tablets, oral [package insert].

[CR43] Tokumo M, Toyooka S, Kiura K, Shigematsu H, Tomii K, Aoe M, Ichimura K, Tsuda T, Yano M, Tsukuda K, Tabata M, Ueoka H, Tanimoto M, Date H, Gazdar AF, Shimizu N (2005). The relationship between epidermal growth factor receptor mutations and clinicopathologic features in non-small cell lung cancers. Clin Cancer Res.

[CR44] Tsao MS, Sakurada A, Ding K, Aviel-Ronen S, Ludkovski O, Liu N, Le Maître A, Gandara D, Johnson DH, Rigas JR, Seymour L, Shepherd FA (2011). Prognostic and predictive value of epidermal growth factor receptor tyrosine kinase domain mutation status and gene copy number for adjuvant chemotherapy in non-small cell lung cancer. J Thorac Oncol.

[CR45] van Zandwijk N, Mathy A, Boerrigter L, Ruijter H, Tielen I, de Jong D, Baas P, Burgers S, Nederlof P (2007). EGFR and KRAS mutations as criteria for treatment with tyrosine kinase inhibitors: retro- and prospective observations in non small-cell lung cancer. Ann Oncol.

[CR46] Zhou C, Wu YL, Chen G, Feng J, Liu XQ, Wang C, Zhang S, Wang J, Zhou S, Ren S, Lu S, Zhang L, Hu C, Hu C, Luo Y, Chen L, Ye M, Huang J, Zhi X, Zhang Y, Xiu Q, Ma J, Zhang L, You C (2011). Erlotinib versus chemotherapy as first-line treatment for patients with advanced EGFR mutation-positive non-small-cell lung cancer (OPTIMAL, CTONG-0802): a multicentre, open-label, randomised, phase 3 study. Lancet Oncol.

